# Investigation on the Attainment of High-Density 316L Stainless Steel with Selective Laser Sintering

**DOI:** 10.3390/ma17010110

**Published:** 2023-12-25

**Authors:** Pengfei Zhu, Xinbo He, Hongda Guan, Zijian Zhang, Tao Zhang, Xuanhui Qu

**Affiliations:** 1 Institute for Advanced Materials and Technology, University of Science and Technology Beijing, Beijing 100083, China; guanhda@163.com (H.G.); zhangzijian8020@163.com (Z.Z.); angiezt@ustb.cn (T.Z.); quxh@ustb.edu.cn (X.Q.); 2 Guangzhou Institute of Advanced Materials, University of Science and Technology Beijing, Guangzhou 510320, China

**Keywords:** selective laser sintering, additive manufacturing, 316L stainless steel, sintering atmosphere, mechanical property

## Abstract

Due to the low density of the green part produced by selective laser sintering (SLS), previous reports mainly improve the sample’s density through the infiltration of low-melting metals or using isostatic pressing technology. In this study, the feasibility of preparing high-density 316L stainless steel using 316L and epoxy resin E-12 as raw materials for SLS combined with debinding and sintering was investigated. The results indicated that in an argon atmosphere, high carbon and oxygen contents, along with the uneven distribution of oxygen, led to the formation of impurity phases such as metal oxides, including Cr_2_O_3_ and FeO, preventing the effective densification of the sintered samples. Hydrogen-sintered samples can achieve a high relative density exceeding 98% without losing their original design shape. This can be attributed to hydrogen’s strong reducibility (effectively reducing the carbon and oxygen contents in the samples, improving their distribution uniformity, and eliminating impurity phases) and hydrogen’s higher thermal conductivity (about 10 times that of argon, reducing temperature gradients in the sintered samples and promoting better sintering). The microstructure of the hydrogen-sintered samples consisted of equiaxed austenite and ferrite phases. The samples exhibited the highest values of tensile strength, yield strength, and elongation at 1440 °C, reaching 513.5 MPa, 187.4 MPa, and 76.1%, respectively.

## 1. Introduction

In today’s industry and society, in order to reduce costs and energy consumption while also achieving better product performance, designers achieve product integration and light weights through structural optimization, which makes parts have complex internal structures, such as complex internal flow channels and overhang structures [1,2,3]. This will undoubtedly bring huge challenges to machining and forming, as traditional forming processes, such as casting, forging, injection molding, have difficulties in preparing complex internal structural components. Additive manufacturing is based on the principle of layer-by-layer stacking to form components, providing a high degree of freedom in material design and manufacturing, and can prepare components of nearly any complex shape [4,5,6]. Therefore, in recent years, additive manufacturing has received increasing attention and research in the preparation of complex metal components.

At present, the representative powder-bed-based additive manufacturing methods entering the field of metal manufacturing include laser powder bed fusion (LPBF) [6,7,8,9] and binder jet printing (BJP) [10,11]. And laser powder bed melting (LPBF) can also be further categorized into two techniques: selective laser melting (SLM) [7,12,13], which mainly uses a fiber laser or YAG laser, and selective laser sintering (SLS), which mainly uses a CO_2_ laser [14,15,16]. These metal additive manufacturing methods can also be divided into two categories: one belongs to direct forming, such as SLM; the other is indirect forming, such as binder jet printing (BJP) and selective laser sintering (SLS), which requires subsequent debinding and sintering to make the parts reach the desired density and strength. Due to the rapid solidification of metals after high-energy laser melting, SLM can make metal parts have fine grains, and thus its mechanical properties may reach the level of forgings [17,18,19], making it a hot research topic in the field of additive manufacturing. However, owing to the rapid solidification (cooling rate can reach 10^5^–10^8^ °C/s), the metal has anisotropy, large internal residual stress, and is prone to deformation and cracking defects [20,21]. There is no internal residual stress problem in the sintered parts of BJP, and BJP has a high forming rate, low cost, and potential for mass production, so it has been widely considered and studied. BJP typically consists of digital model preparation and the printing process, curing process, depowdering process, sintering process, and post-treatment process [20,22,23]. The printing process of BJP is carried out at room temperature, and the properties of already-printed layers during printing are similar to wet clay with low strength that can be easily deformed [24]. As the height direction of the *Z*-axis increases due to gravity, the pressure of the upper powder layers on the lower powder layers gradually increases, resulting in the shift in powder packing density [24]. The inhomogeneity of the internal pressure distribution of the powder bed [24,25,26] will have an impact on some large parts with overhang structures at the bottom, which has a great possibility to cause the distortion, or even fracture, of certain overhang structures. SLS, like BJP, has no residual stress and is isotropic inside the sintered parts. Due to the use of a laser as an energy source to sinter resin binders to bond metal powder particles together, SLS has the characteristic of instant curing. Compared with BJP, SLS has certain advantages in preparing larger-size and complex components with overhang structures. During the printing process, the already-printed layers have a certain strength, which can resist the pressure changes caused by the powder-laying process and the increase in the number of powder layers, reducing the distortion of the printed layer caused by changes in the internal density of the powder bed.

The relative density of the green parts formed with SLS is typically low, often below 40% [27,28,29,30], which poses challenges for achieving densification. As a result, it is commonly used to produce porous structures or for achieving densification through techniques such as low melting point metal infiltration or isostatic pressing technology [14,27,31,32]. Our research focuses on investigating the preparation of high-density metal components through liquid phase sintering, without the need for infiltration and isostatic pressing technology. This approach aims to reduce both the cost and complexity associated with traditional SLS processes for fabricating high-density metal components. In comparison to the costliness and low production rate of SLM direct additive manufacturing, this new SLS indirect additive manufacturing method for fabricating high-density complex metal components offers lower costs and higher production efficiency, making it have great potential for mass production and important value in industrial applications.

In this paper, the binder used was epoxy resin E-12 (in solid state), and the green parts of the 316L stainless steel were fabricated using the selective laser sintering (SLS) technique, followed by debinding and sintering treatments in different atmospheres at different temperatures. The objective of this research was to investigate the feasibility of producing high-density 316L stainless steel components using the SLS method. Additionally, the microstructure and properties of the sintered part were examined.

## 2. Materials and Methods

**Figure 1:**
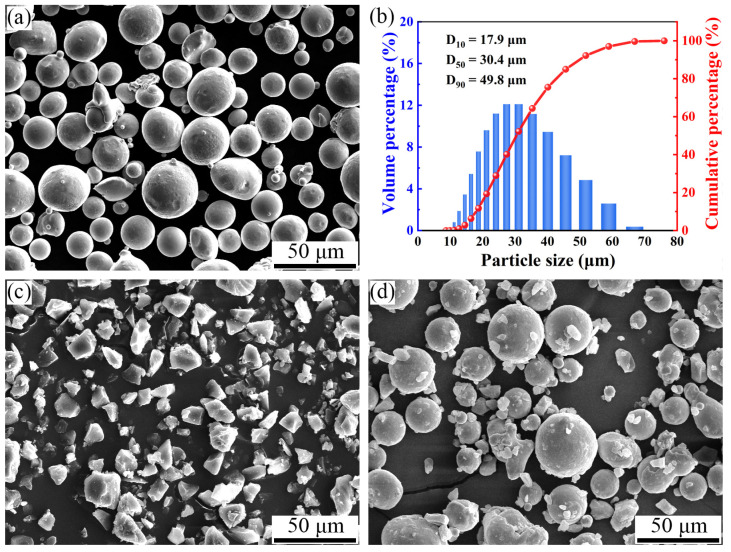
(**a**) SEM image of the 316L powders; (**b**) the powder size distribution of the raw 316L powders; (**c**) SEM image of the E-12 powders; and (**d**) SEM image of the composite powders.

The full process of sample preparation can be mainly divided into three sequential stages: SLS (LPBF), debinding, and sintering. In fact, debinding and sintering are part of the post-treatment process for SLS. Table 1 gives the chemical composition of the as-received 316L stainless steel powders. Figure 1 shows the powder morphology and particle size distribution. The raw materials used in this experiment were near-spherical 316L stainless steel (D_10_ = 17.9 μm, D_50_ = 30.4 μm, D_90_ = 49.8 μm) powders prepared using gas-atomized epoxy resin E-12 (2500 mesh) powders with an irregular shape. The composite powders, consisting of 316L (98.5 wt.%) and E-12 (1.5 wt.%), were mixed in a SZ-3 mixer with a ball-to-powder weight ratio of 2:1 and mixer speed of 43 r/min for 2 h.

**Figure 2:**
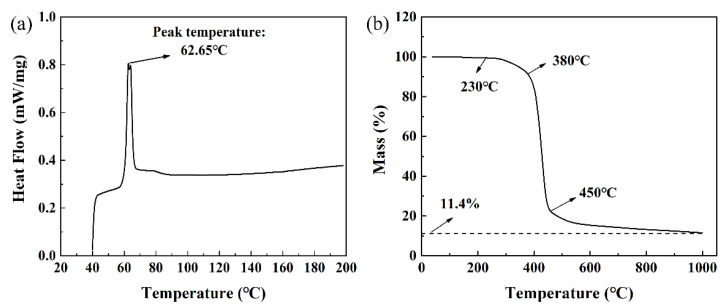
Thermal analysis curves of the E-12 binder: (**a**) DSC; and (**b**) TGA.

The green parts were printed using AFS-300 SLS equipment (Beijing Longyuan AFS Co., Ltd., Beijing, China), and the cuboid green part sizes were 60 mm × 20 mm × 10 mm and 15 mm × 15 mm × 10 mm (length (*X*-axis) × width (*Y*-axis) × thickness (*Z*-axis)). The SLS parameters were set to laser power 12 W, scanning speed 1500 mm/s, scan spacing 0.1 mm, and printing layer thickness 0.1 mm. The preheating temperature of the powder bed was 50 °C, which is usually set to 10 °C below the E-12 softening point (shown in Figure 2a). After printing, the relative density and bending strength of the green parts were 45.3 ± 0.1% and 1.3 ± 0.1 MPa, respectively.

**Figure 3:**
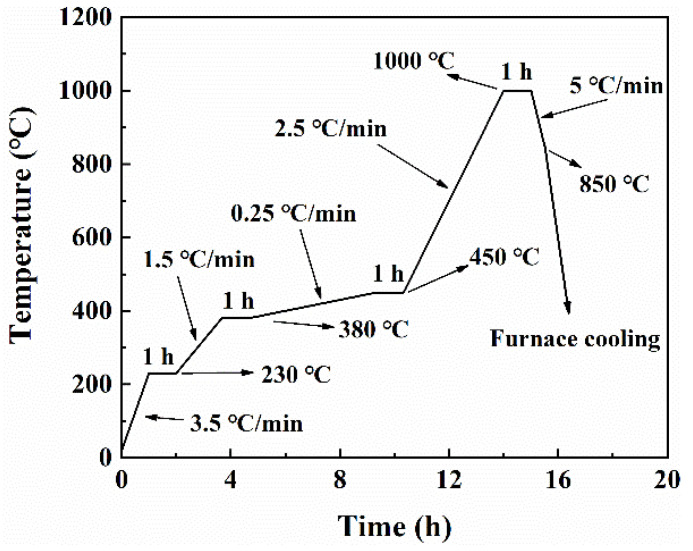
Heating profile of debinding process.

**Figure 4:**
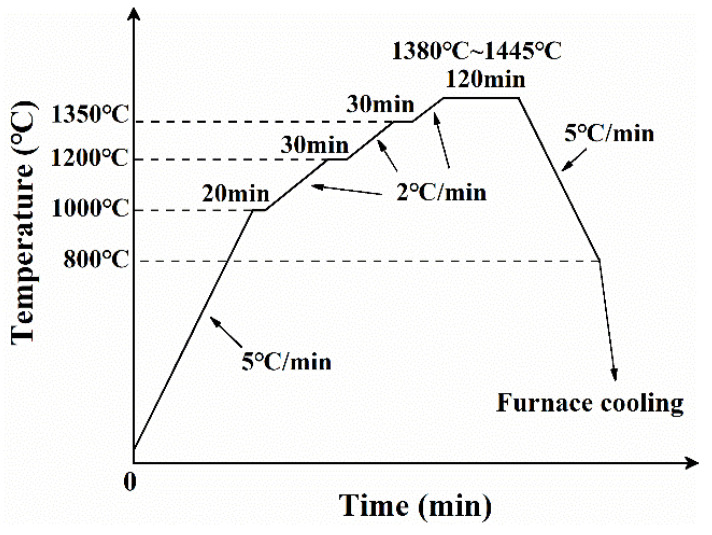
Heating profile of sintering process.

The post-treatment process typically consists of three stages: depowdering (to remove loose powders adhered to the surface of the SLS-printed sample), debinding (to eliminate the binder), and densification treatment (utilizing techniques such as sintering, infiltrating, and isostatic pressure to achieve the desired density and strength) [14,27,31,32]. In this paper, the post-treatment process involved thermal debinding and subsequent high-temperature sintering, both of which were conducted in a tube furnace. After a depowdering process, the green parts underwent debinding in a hydrogen atmosphere. The debinding process (shown in Figure 3) was developed based on the thermal analysis curve of epoxy resin E-12 (shown in Figure 2b). Furthermore, a pre-sintering temperature of 1000 °C was selected as the maximum temperature for debinding. This decision stems from the fact that after debinding at 450 °C, the strength of the debinded part remains low. Consequently, pre-sintering is used to enhance its strength by forming a sintering neck between particles, thereby facilitating subsequent manual handling. Figure 4 displays the sintering heating curve under a hydrogen atmosphere at various temperatures ranging from 1380 °C to 1445 °C. Additionally, argon (Ar) was used as a sintering atmosphere at 1440 °C to investigate its effect on sintering.

The particle size distribution of 316L powders was performed on a laser particle size analyzer (Mastersizer 3000, Malvern Panalytical, Malvern, UK). The microstructure was examined with a metallographic microscope (LEICA DM2700 M, Leica, Wetzlar, Germany) and a scanning electron microscope (LEO-1450, LEO, Oberkochen, Germany). Differential Scanning Calorimeter (DSC) and thermogravimetric analysis (TGA) of epoxy resin E-12 were carried out on a synchronous thermal analyzer (SDT Q600, TA Instruments, New Castle, DE, USA). The oxygen and carbon element contents in the as-received 316L powders and sintered samples were determined using an oxygen/nitrogen/hydrogen elemental analyzer (TCH600, LECO, St. Joseph, MO, USA) and a carbon/sulfur elemental analyzer (EMIA-820V, HORIBA, Kyoto, Japan), respectively. The density of the printed green sample was determined by measuring the mass (using an ME155DU electronic balance with an accuracy of 0.1 mg) and the volume through the sizes of the cuboid (using a Stanley 36-111-23 digital display vernier calipers with an accuracy of 0.01 mm). The sintered density was measured using the Archimedes method. The relative density is obtained by dividing the density of the printed green sample or sintered sample by the theoretical density of 316L. The theoretical density of 316L stainless steel is taken as 7.98 g/cm^3^ [33]. The density measurements were replicated three times to acquire standard error bars. Vickers hardness was performed on a microhardness tester (EM-1500L, Shanghai Hengyi Precision Instument, Shanghai, China) with an applied load of 1000 gf for a duration of 10 s. The bending strength of the green sample was conducted on a microcomputer-controlled electronic universal testing machine (WDW-100, Shandong Wanchen, Jinan, China) with a loading speed of 0.5 mm/min. Tensile properties at room temperature were evaluated using a universal testing machine (WDW-10 E, Jinan Test Gold Group, Jinan, China) with a driving velocity of 1 mm/min.

## 3. Results and Discussion

### 3.1. Microstructure and Properties of 316L Samples Sintered in a Hydrogen (H_2_) Atmosphere

#### 3.1.1. Density, Shrinkage, and Pore Characteristics

**Figure 5:**
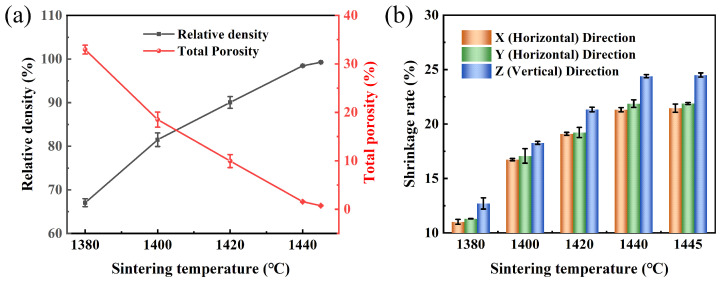
(**a**) Relative density and total porosity; and (**b**) shrinkage rate along different directions of the 316L samples sintered at different temperatures in a H_2_ atmosphere (Z direction is the building direction of printed parts; X/Y direction is perpendicular to the building direction).

As shown in Figure 5, the total porosity decreased and the relative density and shrinkage rate of the sintered samples increased with the rise of the sintering temperature in the range of 1380 °C to 1445 °C. The peak values were achieved at 1445 °C, with a minimum total porosity of 0.7%, maximum relative density of 99.3%, and maximum shrinkage rates in the X, Y, and Z directions being 21.5%, 21.9%, and 24.5%, respectively. Obviously, the vertical direction (Z) exhibited slightly greater shrinkage compared to the horizontal directions (X/Y). This anisotropy of shrinkage during sintering can be attributed to the effect of gravity, which promotes the packing process of powder layers in the vertical direction [34]. Similar findings can also be observed from the binder jet printing (BJP) process [20,35].

**Figure 6:**
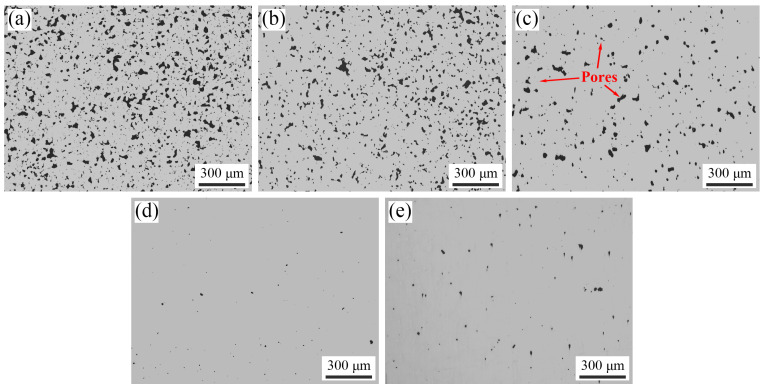
Optical micrographs of the 316L samples sintered in a H_2_ atmosphere at different temperatures: (**a**) 1380 °C, (**b**) 1400 °C, (**c**) 1420 °C, (**d**) 1440 °C, and (**e**) 1445 °C (the pores are shown as the black area).

**Figure 7:**
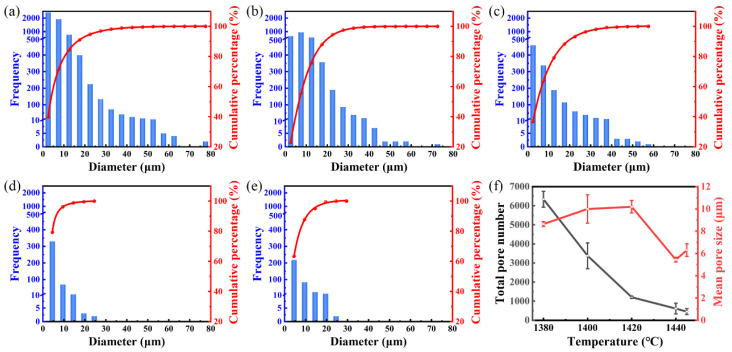
Representative pore size distribution of the 316L parts sintered in a H_2_ atmosphere at (**a**) 1380 °C, (**b**) 1400 °C, (**c**) 1420 °C, (**d**) 1440 °C, and (**e**) 1445 °C; and (**f**) the total pore number and mean pore size of the parts sintered at different temperatures.

The optical micrographs of the 316L sample sintered in a H_2_ atmosphere at different temperatures is shown in Figure 6, with the black region indicating the pores. Image analysis software, Image J, was utilized to make statistics on pore size, and the results are presented in Figure 7. As the sintering temperature increased, interconnected pores (see Figure 6a–c) gradually transformed into isolated ones (see Figure 6d,e), while the pore shape gradually became spherical from an irregular shape. Moreover, the number of pores in each size interval (0~5 μm, 5~10 μm, etc.) decreased, and the larger-sized pores (30~80 μm) gradually reduced and eventually disappeared. When the sintering temperature rose to 1440 °C, the relative density reached 98.5% and the mean pore size reached the minimum of 5.3 μm. However, when the sintering temperature rose to 1445 °C, although the relative density continued to rise to 99.3%, there was a decrease in tiny-sized pores (0~5 μm) accompanied by an increase in larger-sized ones (5~25 μm), leading to an increased mean pore size of 6.3 μm. This abnormal increase in pore size at 1445 °C can be explained as follows: At 1440 °C, the sintering process is in the late sintering stage, the relative density of sintered samples is high, and the gas remaining in the pores is difficult to discharge. At a higher temperature of 1445 °C, the liquid phase content increases and the atomic diffusion ability is enhanced, which promotes grain boundary movement and grain growth, but also promotes the accumulation and growth of tiny pores located in the grain boundaries and grains, resulting in an increase in the mean pore size. In addition, the rise of vapor pressure at higher temperatures may also lead to an increase in pore size [35].

#### 3.1.2. Microstructure

**Figure 8:**
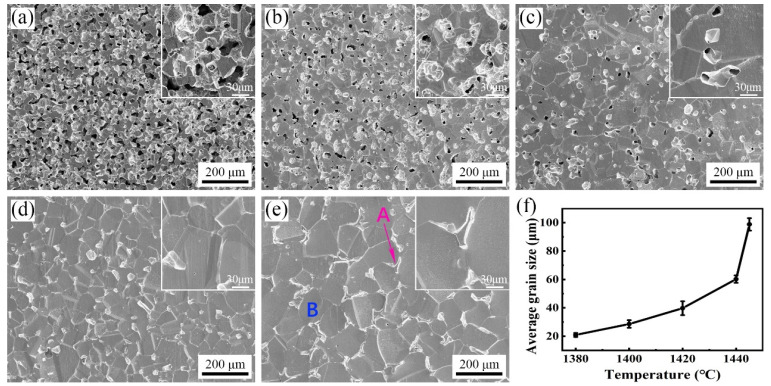
SEM micrographs and grain size of the 316L samples in a H_2_ atmosphere at different sintering temperatures: (**a**) 1380 °C, (**b**) 1400 °C, (**c**) 1420 °C, (**d**) 1440 °C, and (**e**) 1445 °C; and (**f**) average grain size (A denotes location of grain boundary, and B denotes location of grain).

**Figure 9:**
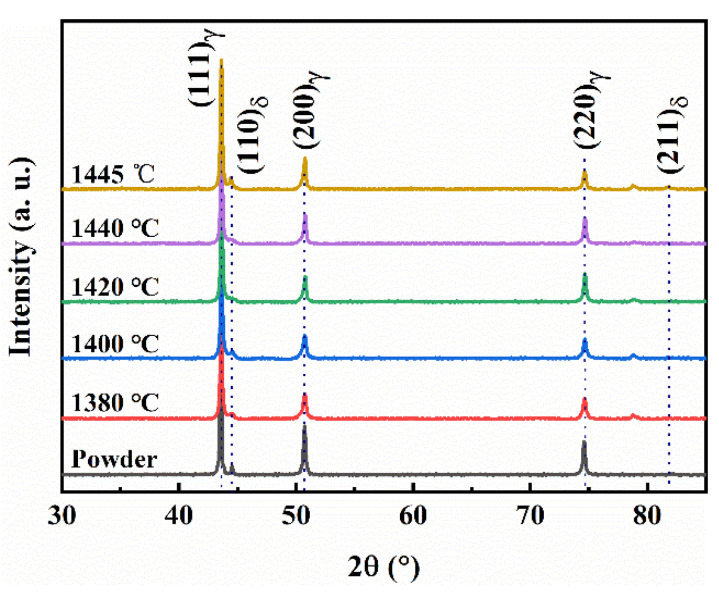
XRD results of the raw 316L powder and sintered parts in a H_2_ atmosphere at different sintering temperatures.

As shown in Figure 8, the microstructure of the sintered 316L stainless steel samples were mainly composed of equiaxed austenite (γ) grains and ferrite (δ) grains located at some austenite grain boundaries, which can be identified with EDS analysis (see Table 2) and an XRD pattern (see Figure 9) of the sintered samples. Cr and Mo elements are generally considered to be ferrite (δ) stabilizing elements, while Ni is an austenitic stabilizing element [36,37]. As shown in Table 2, the content of Cr and Mo in the δ-ferrite phase at the grain boundaries is higher than that in the γ-austenite matrix. In addition, the size of the δ-ferrite phase was relatively large at 1445 °C and can be clearly observed at the grain boundary (see A in Figure 8e), whereas it was difficult to observe within the temperature range of 1380~1440 °C. This phenomenon can be attributed to the high content of liquid phase present at 1445 °C, which promotes both the formation and growth of the δ-ferrite phase.

**Figure 10:**
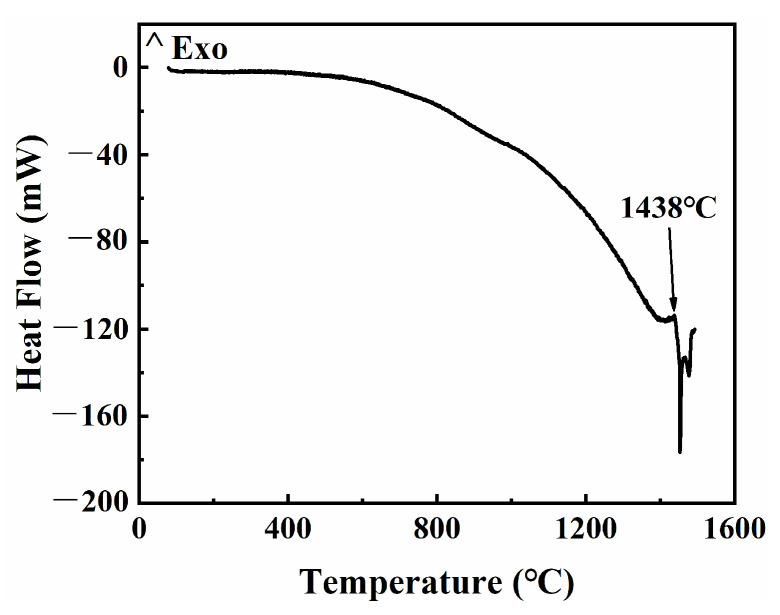
DSC analysis of the raw 316L stainless steel powder.

Figure 8f shows that the grain size increases with the increase in sintering temperature. When the sintering temperature was from 1380 °C to 1420 °C (ΔT = 40 °C), the average grain size increased slowly (20.9 μm → 39.8 μm). As shown in Figure 10, the solidus temperature of the raw 316L powder is 1438 °C, below which it belongs to solid-phase sintering, and the densification process mainly relies on the diffusion of atoms along the grain boundaries. Although the activation energy required for grain growth in this temperature range is low, the grain boundary movement and rapid grain growth are hindered by the existence of a large number of pores [35,38]. When the sintering temperature rose from 1440 °C to 1445 °C (ΔT = 5 °C), the average grain size increased rapidly (60.3 μm → 98.8 μm). This temperature range belongs to liquid-phase sintering, the appearance of the liquid phase not only eliminates most of the pores, but also provides a fast channel for atomic diffusion, which makes the grains have a higher growth rate.

#### 3.1.3. Mechanical Properties

Pore characteristics, such as porosity, pore size, and pore shape, are important factors affecting the mechanical properties of the sintered samples, which typically exhibit incomplete densification [36,39,40,41]. And the pores are potential areas for localized stress concentration and also play an important role in representing the potential sites of the first cracks forming [37,40,42].

**Figure 11:**
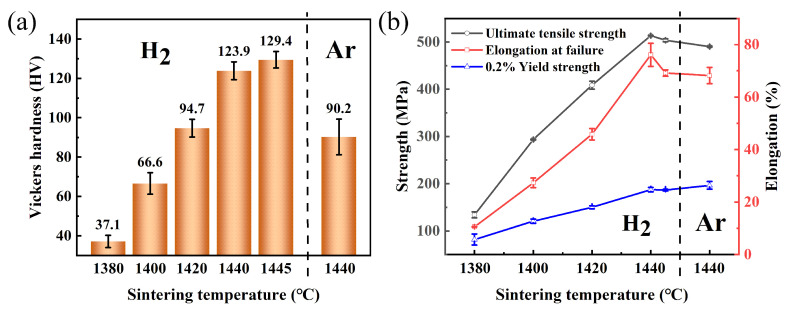
Mechanical properties of the 316L samples in a H_2_ atmosphere at different sintering temperatures and samples in an Ar atmosphere at 1440 °C: (**a**) Vickers hardness; and (**b**) tensile properties.

As shown in Figure 11a, the hardness value increased with the rise in sintering temperature, and the maximum hardness reached 129.4 HV_1_ at 1445 °C. This phenomenon can be attributed to the porosity of the sintered sample, which directly affects its macroscopic hardness. When subjected to the same indenter pressure from the hardness tester, a lower porosity results in a larger volume of matrix that resists pressure, thereby enhancing the resistance of the surface layer against plastic deformation and increasing the hardness of the sintered sample.

**Figure 12:**
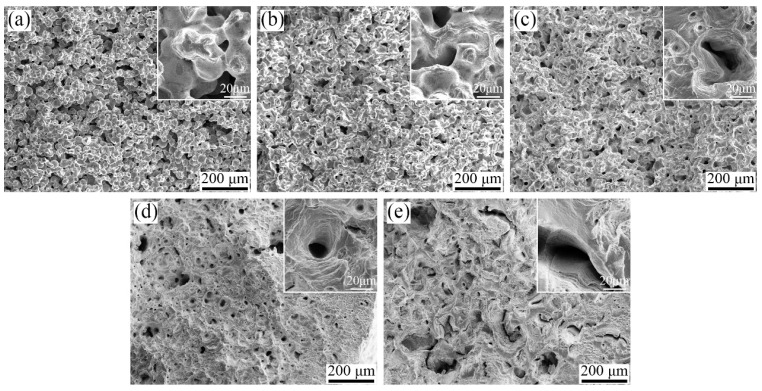
SEM of tensile fracture morphologies of the samples sintered in a H_2_ atmosphere at different temperatures: (**a**) 1380 °C, (**b**) 1400 °C, (**c**) 1420 °C, (**d**) 1440 °C, and (**e**) 1445 °C.

As shown in Figure 11b, the ultimate tensile strength, yield strength, and elongation at failure all increased first and then decreased with the rise in sintering temperature, and reached the maximum values at 1440 °C, which were 513.5 MPa, 187.4 MPa, and 76.1%, respectively. When the temperature was in the range of 1380~1420 °C, a lower relative density (about 67~90%) was observed. As the sintering temperature rises, porosity decreases and the bonding strength between particles strengthens, resulting in increased tensile strength and elongation. Particularly at sintering temperatures of 1380 °C and 1400 °C, the sintering necks and powder particles can be observed clearly on the fracture surface (see Figure 12). At 1440 °C, the sintered sample achieved a high relative density of ~98.5%, significantly reducing porosity and, consequently, grain bonding strength enhanced, leading to the increase in strength and elongation. Notably, deep dimples at the center and parabolic dimples (shallower dimples resulting from higher shear loading) at the edges were distributed across the tensile fracture surface at this temperature. When the sintering temperature rose to 1445 °C, although the porosity was further reduced (relative density of ~99.3%), the strength and elongation at this temperature were slightly lower than that at 1440 °C. This abnormal phenomenon is associated with pore size and pore shape. As shown in Figure 6 and Figure 7 above, the sample sintered at 1440 °C exhibited a tiny pore size (~5.3 μm) with a nearly spherical shape. In contrast, the sample sintered at 1445 °C displayed an increased pore size (~6.3 μm) along with a higher degree of irregularity in pore shape. The larger pore sizes and more irregular shapes are prone to inducing stress concentration and fracture, consequently diminishing the strength of the sintered sample [39,40,43]. Moreover, compared with 1440 °C, a change in fracture morphology can be observed, with the presence of a combination of cracks, parabolic dimples, and deep dimples on the surface of the tensile fracture [36,44].

### 3.2. Effect of the Sintering Atmosphere on the Microstructure and Mechanical Properties of the Sintered Samples

**Figure 13:**
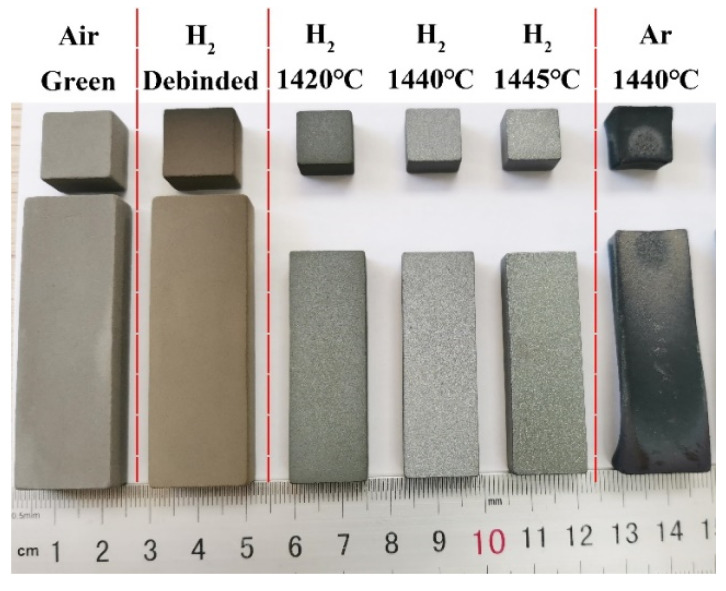
Images of the green part, debinded part, and samples sintered at different temperatures in different atmospheres.

**Figure 14:**
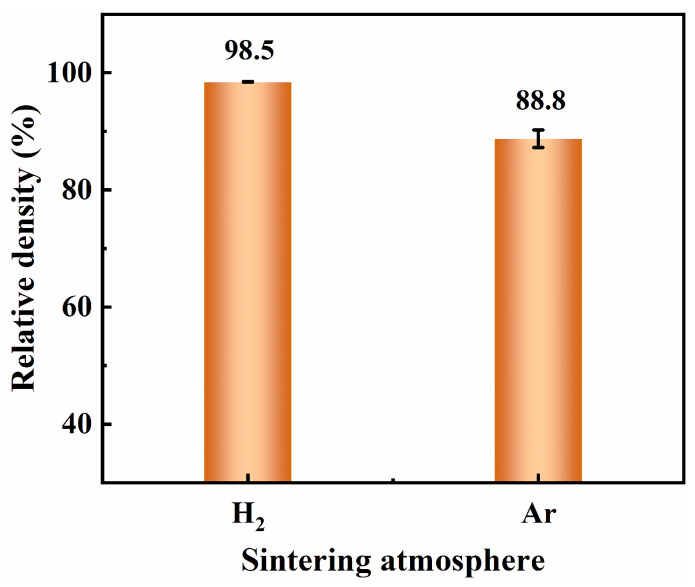
Relative density of the 316L samples sintered at 1440 °C in different atmospheres.

**Figure 15:**
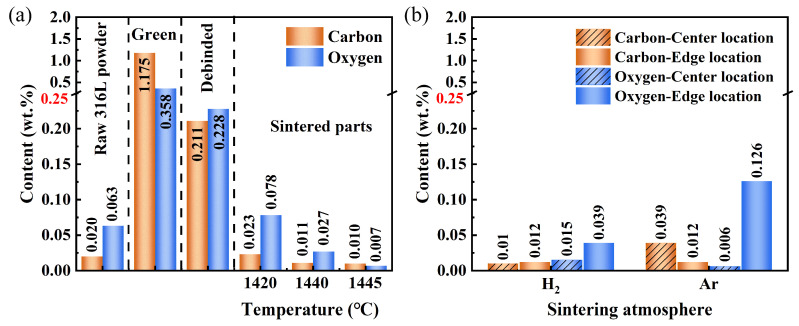
(**a**) Carbon and oxygen contents of the green part, debinded part, and samples sintered in a hydrogen (H_2_) atmosphere at different temperatures; and (**b**) carbon and oxygen contents at the center and edge locations of the 316L samples sintered at 1440 °C in different atmospheres.

Figure 13 shows the images of the green part, debinded part, and sintered samples at different temperatures in different atmospheres. The hydrogen-sintered sample exhibited uniform shrinkage and maintained its original design shape at different sintering temperatures, ultimately achieving a relative density of 98.5% at 1440 °C (see Figure 14). Conversely, the argon-sintered sample experienced severe distortion of the design shape, resulting in a relative density of only 88.8% at 1440 °C. Figure 15 presented the carbon and oxygen contents of the green part, debinded part, and sintered samples at different temperatures in different atmospheres. The raw 316L powders had a carbon content of 0.020 wt.% and an oxygen content of 0.063 wt.%. After debinding, the carbon and oxygen contents of the sample rose to 0.211 wt.% and 0.228 wt.%, respectively. This rise may be attributed to metal oxidation during the mixing and printing processes, as well as residual carbon generated after the debinding treatment. However, excessive carbon and oxygen contents will hinder the densification process during the powder metallurgy samples’ sintering [45,46].

Figure 15b shows the carbon and oxygen contents at different locations when the sintering temperature was 1440 °C. It can be observed that the argon-sintered sample exhibited significantly higher levels of carbon and oxygen contents compared to the hydrogen-sintered sample, as argon (Ar) is an inert gas with no deoxygenation or decarbonization capacity. Moreover, there was a big variation in carbon and oxygen contents in different locations of the argon-sintered sample, particularly for oxygen. Specifically, the center location had an oxygen content of 0.006%, while the edge location displayed an oxygen content of 0.126%, indicating a difference of two orders of magnitude. The excessive residual carbon and oxygen contents, along with their uneven distribution, severely impact the sintering process, leading to severe distortion of the design shape in the argon-sintered sample (see Figure 13). Consequently, its relative density is low, reaching only 88.8%. Notably, the average carbon and oxygen contents in the argon-sintered sample were measured as 0.026 wt.% and 0.066 wt.%, respectively—significantly lower than those found in the debinded sample (0.211 wt.% for carbon and 0.228 wt.% for oxygen). This is because the residual carbon after debinding can react with the oxygen in 316L during the sintering process, resulting in a reduction in both the carbon and oxygen contents within the sintered sample. The chemical reaction equations are as follows [47,48]:

b C + Me_a_O_b_ ⇋ b CO + a Me (1)

b CO + Me_a_O_b_ ⇋ a Me + b CO_2_ (2)

where Me_a_O_b_ represents metal oxide, while Me denotes a metallic element.

As shown in Figure 15a, when hydrogen was used as the sintering atmosphere, the contents of carbon and oxygen in the sintered sample decreased with the increase in sintering temperature. When the temperature reached 1440 °C, the contents of carbon and oxygen in the sample were 0.011 wt.% and 0.027 wt.%, respectively, which were lower than that measured in the raw 316L powder (0.20 wt.% for carbon and 0.063 wt.% for oxygen). Furthermore, Figure 15b illustrates a relatively uniform distribution of carbon and oxygen contents between the center and edge locations. These findings indicate that hydrogen, as a reducing atmosphere, exhibits excellent decarbonization and deoxidation capabilities, and eliminates the excess carbon and oxygen generated by the addition of a resin binder and the mixing and printing processes. Here, apart from carbon serving as a reducing agent for the reduction reactions described by Equations (1) and (2), deoxidation reactions (3) and (4) [47,49,50] and decarbonization reactions (5) using hydrogen (H_2_) as a reducing agent may also occur:

b H_2_ + Me_a_O_b_ ⇋ a Me + b H_2_O (3)

H_2_ + CO_2_ ⇋ H_2_O + CO (4)

2 H_2_ + C ⇋ CH_4_ (5)

**Figure 16:**
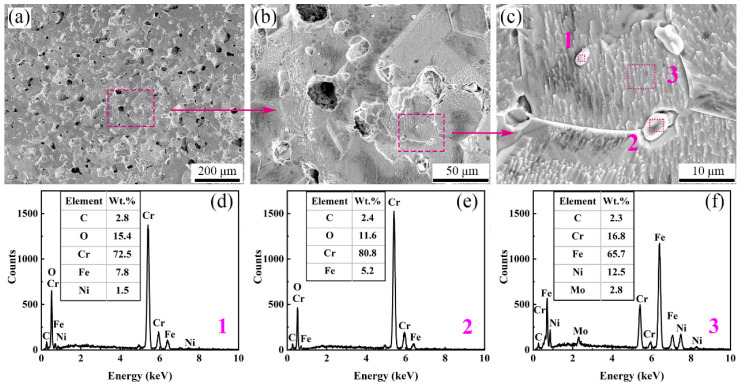
SEM micrographs and EDS analyses of the 316L samples in an Ar atmosphere at 1440 °C: (**a**) ×200; (**b**) ×1000; (**c**) ×5000; and (**d**) EDS result at point 1 in (**c**); (**e**) EDS result at point 2 in (**c**); (**f**) EDS result at point 3 in (**c**).

**Figure 17:**
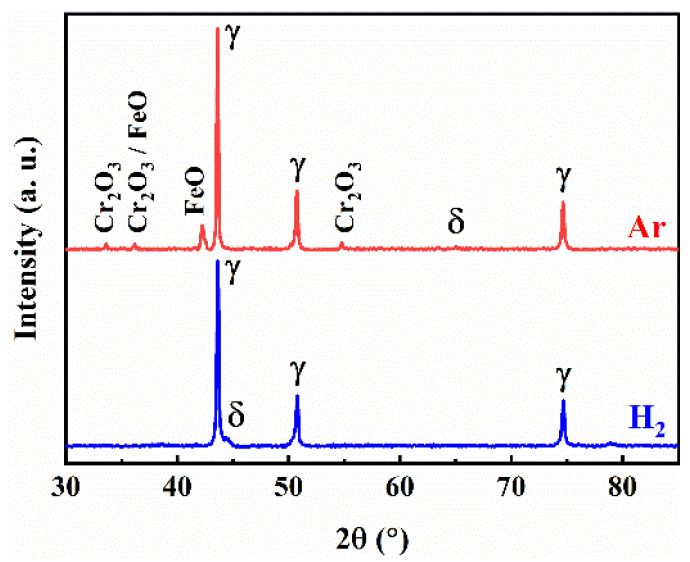
XRD results of the sintered parts in different sintering atmospheres at 1440 °C.

As shown in Figure 16, both within the grains and at the grain boundaries of the argon-sintered sample, near-spherical or short rod-like impurity phases can be observed. The oxygen (O) and chromium (Cr) contents in these second phases (points 1 and 2) were significantly higher than those in the matrix (point 3). And, based on the corresponding XRD results (Figure 17), it can be deduced that metal oxides Cr_2_O_3_ and FeO emerged in the microstructure of the argon-sintered sample. Since XRD analysis cannot detect phases with very low content, various impurity phases (such as oxides or carbides), including but not limited to Cr oxides and Fe oxides, may appear in the argon-sintered sample. The densification of the powder metallurgy parts relies on atomic diffusion, with the grain boundary serving as a crucial pathway for atomic migration. Most of the pores are located at the grain boundaries (see Figure 8 and Figure 16). The movement and growth of grain boundaries change the shape and size of pores, facilitating their reduction and elimination [51]. However, impurity phases predominantly reside at the grain boundary (see Figure 16), exerting a pinning effect [52,53] that hinders grain boundary movement. Consequently, this will impede the pores’ reduction and elimination, ultimately preventing the effective densification of the sintered sample and even possibly resulting in uneven shrinkage deformation. Conversely, when sintering in a hydrogen (H_2_) atmosphere, no noticeable impurity phases were observed within both the grain boundaries and the grains (see Figure 8).

**Figure 18:**
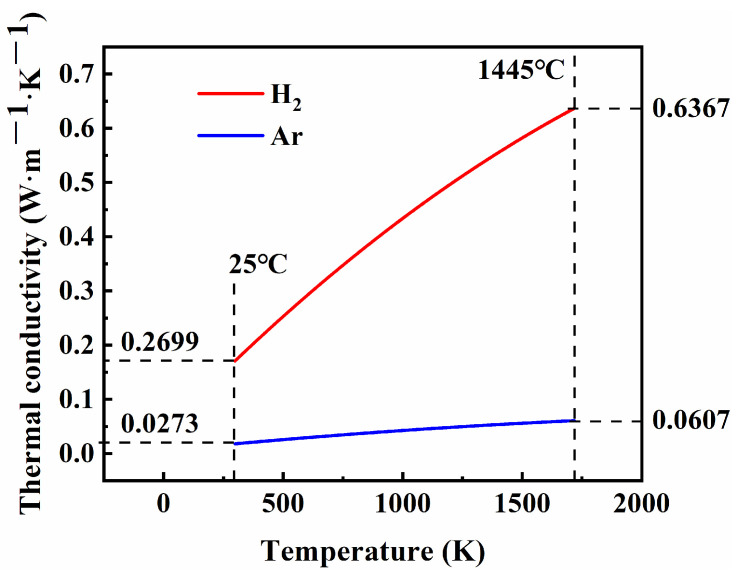
Thermal conductivities of the sintering atmospheres of hydrogen (H_2_) and argon (Ar) at different temperatures.

In addition, the uniformity of temperature in the tube furnace also affects the sintering process. A previous study for metal powder injection molding (MIM) had demonstrated that local temperature differences can lead to uneven shrinkage in the sintering process [54]. As shown in Figure 13, the shrinkage at both ends of the argon-sintered sample was significantly smaller than that observed at the middle position. The thermal conductivity formula of gas is shown as follows [55]:

k_G_ = A + B T + C T^2^ (6)

where k_G_ denotes the thermal conductivity of the gas (W·m^−1^·K^−1^); T denotes the thermodynamic temperature (K); and A, B, and C denote regression coefficients, which vary according to the type of gas. For argon, these coefficients A, B, and C are 0.00548, 4.3869 × 10^−5^, and −6.8141 × 10^−9^, respectively; whereas for hydrogen they are 0.03951, 4.5918 × 10^−4^, and −6.4933 × 10^−8^, respectively. Figure 18 illustrates the thermal conductivities of argon and hydrogen at different temperatures based on Equation (6). It can be observed that within the temperature range of 25 °C to 1445 °C, hydrogen (H_2_) exhibits a thermal conductivity approximately ten times higher than that of argon (Ar). Obviously, hydrogen (H_2_), as a sintering atmosphere, offers superior thermal conductivity compared to argon (Ar), thereby reducing the temperature gradient in the tube furnace and improving the internal thermal uniformity of the sintering sample, which is conducive to the sintering densification process.

The mechanical properties of the argon-sintered sample at 1440 °C are presented in Figure 11 above. As shown in Figure 11a, the Vickers hardness of the argon-sintered sample (90.2 HV_1_) was considerably lower than that of the hydrogen-sintered sample (123.9 HV_1_) at the same sintering temperature of 1440 °C, and slightly lower than that of the hydrogen-sintered sample (94.7 HV_1_) at 1420 °C. This can be attributed to the relative density of the argon-sintered sample being 88.8% (see Figure 14), which is lower than that observed for the hydrogen-sintered sample (98.5% at 1440 °C and 90.1% at 1420 °C). As shown in Figure 11b, both the ultimate tensile strength and the elongation at failure of the argon-sintered sample were lower compared to those of the hydrogen-sintered sample at the same sintering temperature (1440 °C), but higher than that of the hydrogen-sintered sample at 1420 °C with higher density. And it should be mentioned that the yield strength of the argon-sintered sample was higher than that of hydrogen-sintered sample, with comparable or higher densification. This phenomenon can be attributed to the emergence of numerous impurity phases at both grain boundaries and within grains in the sintered samples (see Figure 16). These impurity phases effectively hinder the movements of grain boundaries or dislocations during plastic deformation, thereby significantly enhancing both the strength and elongation properties of the sintered samples.

## 4. Conclusions

In this study, 316L stainless steel green samples were produced using selective laser sintering with gas-atomized 316L powders and epoxy resin E-12 powders as raw mate-rials. Subsequently, the densification of the SLS-ed samples was realized by adjusting the sintering temperature (1380~1445 °C) and sintering atmosphere (Ar and H_2_) after debinding, followed by an investigation into the microstructure and properties. The main conclusions can be drawn as follows:

(1) The argon-sintered sample exhibited a limited relative density of 88.8% and experienced significant distortion from its original design shape when sintered at a temperature of 1440 °C. This can be primarily attributed to the high carbon and oxygen contents present in the sample, as well as their uneven distribution, particularly the non-uniform distribution of the oxygen content. Consequently, impurity phases such as metal oxides (Cr_2_O_3_ and FeO) were formed, preventing grain boundary movement and the elimination of pores, thus hindering the sintering densification process;
(2) The hydrogen-sintered sample can achieve a high relative density exceeding 98% at 1440 °C and 1445 °C, while successfully maintaining the original design shape. The results can be attributed to two factors: firstly, due to hydrogen’s strong reducibility, it effectively diminished both its carbon and oxygen contents, improved their distribution uniformity, and eliminated the hindrance of impurity phases such as metal oxides to the sintering process; secondly, within the temperature range of 25~1445 °C, hydrogen (H_2_) exhibits approximately ten times higher thermal conductivity compared to argon (Ar), which will reduce temperature gradients within the sintered sample and promote an efficient sintering process;
(3) The microstructure of the hydrogen-sintered sample consisted of equiaxed austenite and ferrite phases. The printed green part had a relative density of 45.3%, while a maximum relative density of 99.3% can be obtained when the sample is sintered at 1445 °C in a H_2_ atmosphere. The shrinkage of the sintered sample exhibited anisotropy. The hydrogen-sintered sample at 1440 °C exhibited the best comprehensive mechanical properties, with Vickers hardness, tensile strength, yield strength, and elongation values of 123.9 HV_1_, 513.5 MPa, 187.4 MPa, and 76.1%, respectively. Simultaneously, the corresponding relative density reached 98.5%, and the shrinkage rates in three directions were 21.3% (X), 21.9% (Y), and 24.4% (Z).

## Figures and Tables

**Table 1:** Chemical composition of the as-received 316L stainless steel powders.

Element	Cr	Ni	Mo	Si	Mn	C	O	S	P	Fe
Content (wt.%)	17.05	10.76	2.47	0.42	0.78	0.020	0.063	0.004	0.020	Bal.

**Table 2:** Composition (wt.%) measured with EDS of the areas highlighted in Figure 8e. The result is the mean value obtained from measurements taken at ten different locations of grain (G) and grain boundaries (GB).

Location	Phase	Cr	Mo	Ni	Fe
A (GB)	δ-ferrite	25.7 ± 0.5	4.8 ± 0.3	5.3 ± 0.4	63.5 ± 0.6
B (G)	γ-austenite	17.3 ± 0.6	2.2 ± 0.1	13.1 ± 0.8	66.8 ± 0.8

## Data Availability

Data will be made available on request.
